# Investigation on the interface between Li_10_GeP_2_S_12_ electrolyte and carbon conductive agents in all-solid-state lithium battery

**DOI:** 10.1038/s41598-018-26101-4

**Published:** 2018-05-23

**Authors:** Kyungho Yoon, Jung-Joon Kim, Won Mo Seong, Myeong Hwan Lee, Kisuk Kang

**Affiliations:** 1 Department of Materials Science and Engineering, Seoul National University, 1 Gwanak-ro, Gwanak-gu, Seoul, 151-742 Republic of Korea; 2 Samsung SDI, Samsung-ro 130, Yeongton-gu, Suwon-si, Gyeonggi-do, 16678 Republic of Korea; 3 Center for Nanoparticle Research at Institute for Basic Science (IBS), Seoul National University, 1 Gwanak-ro, Gwanak-gu, Seoul, 08826 Korea

## Abstract

All-solid-state batteries are considered as one of the attractive alternatives to conventional lithium-ion batteries, due to their intrinsic safe properties benefiting from the use of non-flammable solid electrolytes in ASSBs. However, one of the issues in employing the solid-state electrolyte is the sluggish ion transport kinetics arising from the chemical and physical instability of the interfaces among solid components including electrode material, electrolyte and additive agents. In this work, we investigate the stability of the interface between carbon conductive agents and Li_10_GeP_2_S_12_ in a composite cathode and its effect on the electrochemical performance of ASSBs. It is found that the inclusion of various carbon conductive agents in composite cathode leads to inferior kinetic performance of the cathode despite expectedly enhanced electrical conductivity of the composite. We observe that the poor kinetic performance is attributed to a large interfacial impedance which is gradually developed upon the inclusions of the various carbon conductive agents regardless of their physical differences. The analysis through X-ray Photoelectron Spectroscopy suggests that the carbon additives in the composite cathode stimulate the electrochemical decomposition of LGPS electrolyte degrading its surface during cycling, indicating the large interfacial resistance stems from the undesirable decomposition of the electrolyte at the interface.

## Introduction

A development of safe and reliable energy storage has been re-highlighted with the recent incidents involving battery swelling/burning and subsequent recall of the lithium ion batteries^1,2^. In particular, with the lithium-ion battery technology being actively incorporated into electric vehicles and large-scale energy storage systems, the safety of the large-size batteries cannot be overstated. The organic liquid electrolyte used in conventional lithium-ion batteries usually acts as a fuel for the combustion in a thermal-runaway reaction, thereby leading to safety incidents^3^. On the other hand, ASSBs exploit non-flammable solid electrolytes, making them much more tolerant to reactions with such explosive natures, thus are regarded as the promising safe alternative to the current lithium-ion batteries^4–6^. However, employing solid-state electrolyte in the ASSBs accompanies several important technical issues that need to be addressed such as the necessity of finding solid-state fast ionic conductors whose ionic conductivity should be comparable to that of the liquid electrolyte and the careful interface control of the solid-solid contacts among the various components in the battery^7–9^.

Extensive search for novel solid electrolytes with high ionic conductivity has been carried out for the past decades in the development of ASSBs^8,10–12^. Among them, recent discovery of sulfide-based electrolyte including LGPS has invigorated the field, as the ionic conductivity could reach the similar level to that of the commercial organic liquid electrolytes^8^. Moreover, the malleable mechanical nature of the sulfides generally offer a good physical contact with other components in the electrode, alleviating the unnecessarily large contact resistance between the solid components^13^. Notable is that typical oxides-based solid electrolytes generally require a high-temperature sintering to reduce the abnormally large contact resistance, thus offering limited options for cell configurations^14–19^. Nevertheless, the construction of reliable ASSBs using sulfide electrolytes still faces several technical challenges^20–22^. One of the critical bottlenecks is to effectively maintain the interface stability between the electrode and the solid electrolyte in terms of not only the physical contact but also the chemical stability. For example, S. Wenzel *et al*. reported that chemical reactions at Li/LGPS interface leads to the decomposition of LGPS, producing an undesirable interphase composed of Li_3_P, Li_2_S, and Li-Ge alloy^20^. A. Sakuda *et al*. also revealed that an interfacial layer was formed after initial charge of the battery due to the mutual diffusion of Co, P, and S at the interface between LiCoO_2_ and Li_2_S-P_2_S_5_^21^. In this regard, various surface modifications and protections of electrode materials in ASSBs have been conducted in order to decrease the side reaction at the interface with the solid electrolyte^23–28^.

In a practical cell configuration, conductive agents are inevitable in the electrode fabrication to aid in the electronic conduction throughout the electrode. In ASSBs, thus, not only the interface between the solid electrolyte and the electrode material, but also that with the conductive agents have to be carefully investigated. While several studies showed that ASSBs based on LGPS electrolyte without carbon additives could exhibit first discharge capacity near the theoretical capacity of LiCoO_2_ with relatively stable cycle life^29^, such electrochemical cycling was possible because LiCoO_2_ becomes metallic after delithiation, and was examined at relatively slow current rate (~0.1 C)^8,29,30^. In order to achieve reasonably high power density and for the adoption of other cathode materials that are generally not metallic in ASSBs, the inclusion of conductive agents such as carbon additives in composite cathodes is unavoidable. Moreover, one of the recent studies by Han *et al*. further motivated us to study on the interface between the solid electrolyte and the conductive carbon in ASSBs^31^. In their work, Han *et al*. utilized the oxidation and reduction of LGPS as an active electrode materials by preparing the composite cathode by mixing LGPS with a large amount of carbon black^31^. While the reversible electrochemical activity of LGPS in ASSBs is interesting and rather unexpected, it strongly implies that the inclusion of carbon additives seems to stimulate the electrochemical activity of LGPS when a sufficient amount of carbon surrounds the LGPS. Furthermore, it suggests that the nature of LGPS in conventional carbon-containing composite cathodes would vary with electrochemical cycling, which has not been considered in the evaluation of the cathode performance. In this respect, here we carefully study the effect of carbon additives in ASSBs based on LGPS electrolyte. It is found that the inclusion of carbon conductive agents regardless of their physical differences such as carbon nanoparticles and carbon nanotubes in composite cathode results in the inferior performance of the cathode in comparison to that without the carbon additives. The origin of the poor performance is observed to be the large interfacial impedance which is developed during the initial cycling processes, which is related with the undesirable decomposition of the electrolyte at the interface with the carbon.

## Results

**Figure 1:**
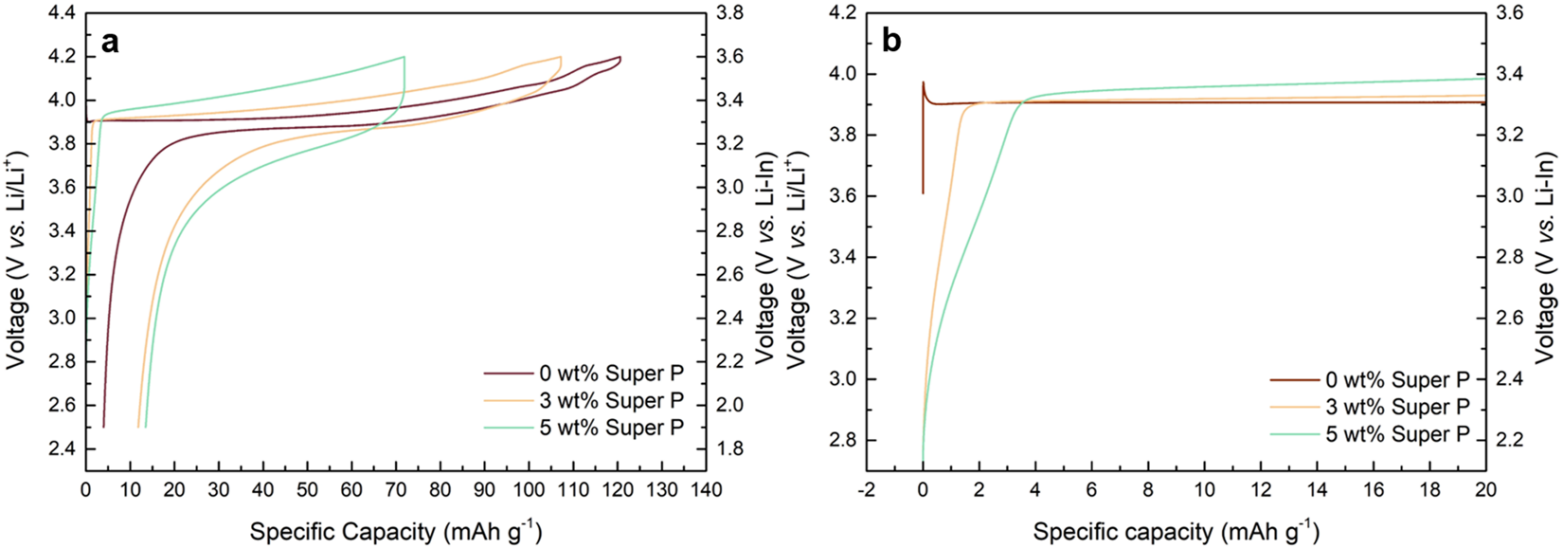
First charge/discharge profile of ASSB (Li-In/LGPS/LCO) with various wt% of Super P (carbon additive) in composite cathode. (**a**) First galvanostatic discharge/charge profile of ASSB (Li-In/LGPS/LCO). (**b**) Magnification of first charge profile in the range of −2~20 mAh g^−1^. All galvanostatic tests were carried out at 0.075 C.

**Figure 2:**
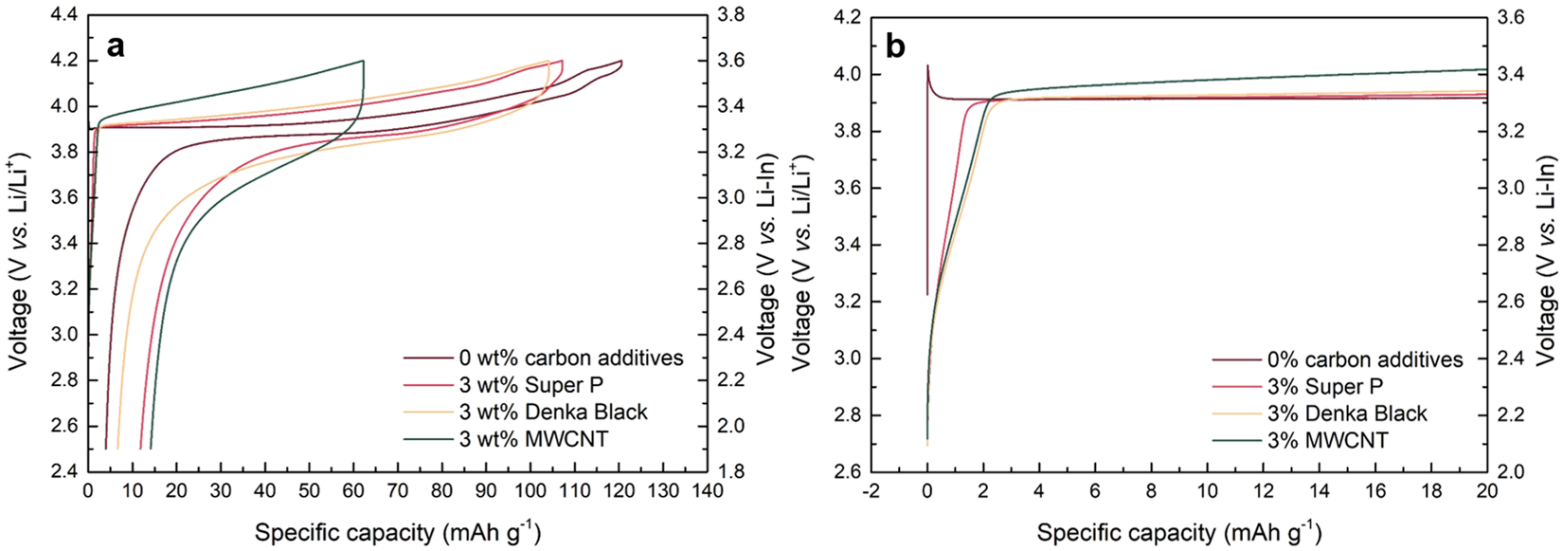
First charge/discharge profile of ASSB (Li-In/LGPS/LCO) with different types of carbon additives in composite cathode. (**a**) First galvanostatic discharge/charge profile of ASSB (Li-In/LGPS/LCO). (**b**) Magnification of first charge profile in the range of −2~20 mAh g^−1^. All galvanostatic tests were carried out at 0.075 C.

Charge/discharge profiles of composite cathodes comprising of LiCoO_2_, LGPS and different ratios of carbon nanoparticles (Super P: 0, 3, and 5 wt%) were first examined in the galvanotactic mode at 0.075 C as shown in Fig. 1a. The composite cathode without Super P carbon (red line) exhibits a typical charge/discharge profile of LiCoO_2_ with a plateau at 3.9 V *vs*. Li/Li^+^ at the given operating voltage range^32,33^. However, the inclusion of Super P in the composite results in the substantial reduction of the capacity and larger polarization between the charge and discharge despite the expectedly enhanced electrical conductivity with the added conductive agents. More careful examination of the charge profiles in Fig. 1b reveals that there appears an unusual slope region in the beginning of the charging, which increases with the amount of the carbon added. The composite cathode with 3 wt% Super P shows the slope region with a capacity equivalent to about 1.5 mAh g^−1^, and that with 5 wt% Super P exhibits a longer slope with approximately 3.5 mAh g^−1^, which indicates that even small change in the carbon amount sensitively influences on the cathode performance. It should be noted that a similar slope region during the charging has been also observed in several previous reports adopting carbon in the ASSBs employing LGPS^34,35^. We could also confirm that such electrochemical behavior is universally observed regardless of the carbon type as shown in Fig. 2. Employing other type of carbon nanoparticles such as Denka black or carbon nanotubes such as MWCNT consistently show the notable reduction in the capacity along with the larger polarization in comparison to the carbon-free cathode (See Figure S3 for the material characterization). Moreover, the cathode with MWCNT undergoes more serious degradation with a same content of carbon, which is attributed to the one-dimensional shape of MWCNT that can effectively promote the electrical network in the composite^36–38^. It hints that the undesirable side reactions that deteriorate the cathode performance are more easily triggered by the facile electrical supply in the cathode. Concerning the origin of the unusual slope from the composite cathode, it is worthwhile to note that the voltage of a pseudo-plateau in the slope begins around 2.6 V *vs*. Li/Li^+^, which is similar to the voltage where LGPS oxidation occurs with a large amount of carbon (25 wt%) as reported in a previous research^28^. It strongly suggests that the slope region in the initial charging step is a signature of the electrochemical activation and/or the subsequent degradation of LGPS, which will be discussed in more detail later.

**Figure 3:**
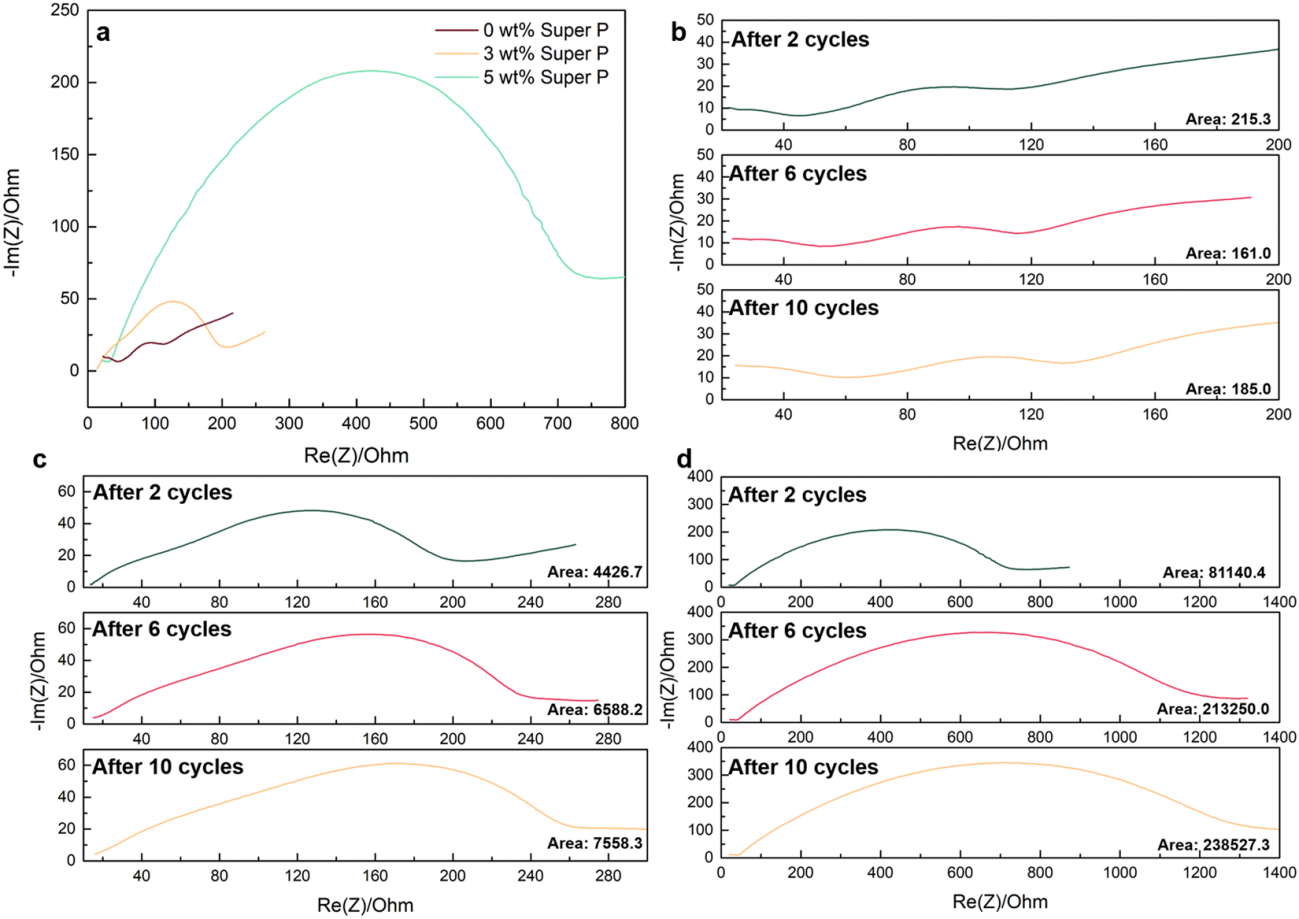
Electrochemical impedance spectrum of ASSB after 10 cycles at 0.075 C with various weight ratios of Super P. (**a**) Electrochemical impedance spectrum of ASSB after 2 cycles at 0.075 C with various weight ratios of Super P. (**b**) Impedance spectrum of 0 wt% Super P after 2, 6 and 10 cycles. (**c**) Impedance spectrum of 3 wt% Super P after 2, 6 and 10 cycles. (**d**) Impedance spectrum of 5 wt% Super P after 2, 6 and 10 cycles. Areas of semi-circles are calculated and displayed on the figure. All electrochemical impedance tests were performed in room temperature.

In order to further understand the effect of carbon additives to the electrochemical properties, EIS analysis was carried out for composite cathodes with different mass fraction of Super P after two electrochemical cycling of them. Figure 3a clearly shows that the impedance of the composite cathode increases with the amount of the Super P added, while the carbon-free cathode shows the lowest value. It suggests that the reduction of the capacity displayed in Fig. 1 is primarily due to the systematic increase in the total impedance with the conductive carbon agents. In addition, the EIS analysis was further performed for the composite cathodes after extended cycle numbers (6 and 10 cycles). Figure 3b–d shows that the increase in the impedance is notably faster with the higher contents of carbon. While the carbon-free composite cathode exhibits negligible change in the total impedance as cycle number increases in Fig. 3b, those with 3 and 5 wt% Super P (Fig. 3c,d) not only display larger total impedance but also greater increase of it with cycle, particularly during the initial a few cycles (~6 cycles). Our attempts to quantitatively analyze the precise factors in the impedance using several equivalent circuit models have failed due to the ambiguity in assigning interfaces such as In/LGPS, carbon/LGPS and LiCoO_2_/LGPS^24^. Instead, concerning the speculation of the electrochemical activation and the degradation of LGPS at the interface with carbon, we compared the areas under the semi-circle at ~100 Hz in the EIS spectra of each samples as denoted as insets in each figure, which are typically attributed to the interfacial resistance at a cathode interface^24^. The comparison clearly reveals that the increase in the area of semi-circle for 5 wt%-containing sample is much greater than that of the 3 wt% one, while that of the carbon-free sample remains unchanged, supporting that the interface has been degraded with the inclusion of the carbon additives during the electrochemical cycles. In addition, EIS analysis for ASSB with 5 wt% Super P without current being applied was carried out as a function of time in order to clarify whether the degradation is a spontaneous chemical reaction. Figure S5 shows that the change in total impedance of ASSB after rest time is negligible with the absence of the large semi-circle at ~100 Hz, which confirms that the large interfacial resistance originates the electrochemical reaction, not from chemical reaction.

Such large interfacial impedance may originate from various factors such as non-uniform current distribution and poor physical contact between electrode material and electrolyte^13,39,40^. However, since carbon additives usually offer better current distribution in a cathode matrix, it is unlikely that the presence of carbon additives would increase the total impedance of composite cathode simply by inducing non-uniform current distribution^39^. Furthermore, a significant alternation in the physical contact between electrolyte and electrode arising from the 5 wt% carbon presence seems not plausible, especially when the ratio between LGPS and LiCoO_2_ is fixed to 7:3, and LGPS is known to exhibit good interface contact due to their malleable property^13^.

**Figure 4:**
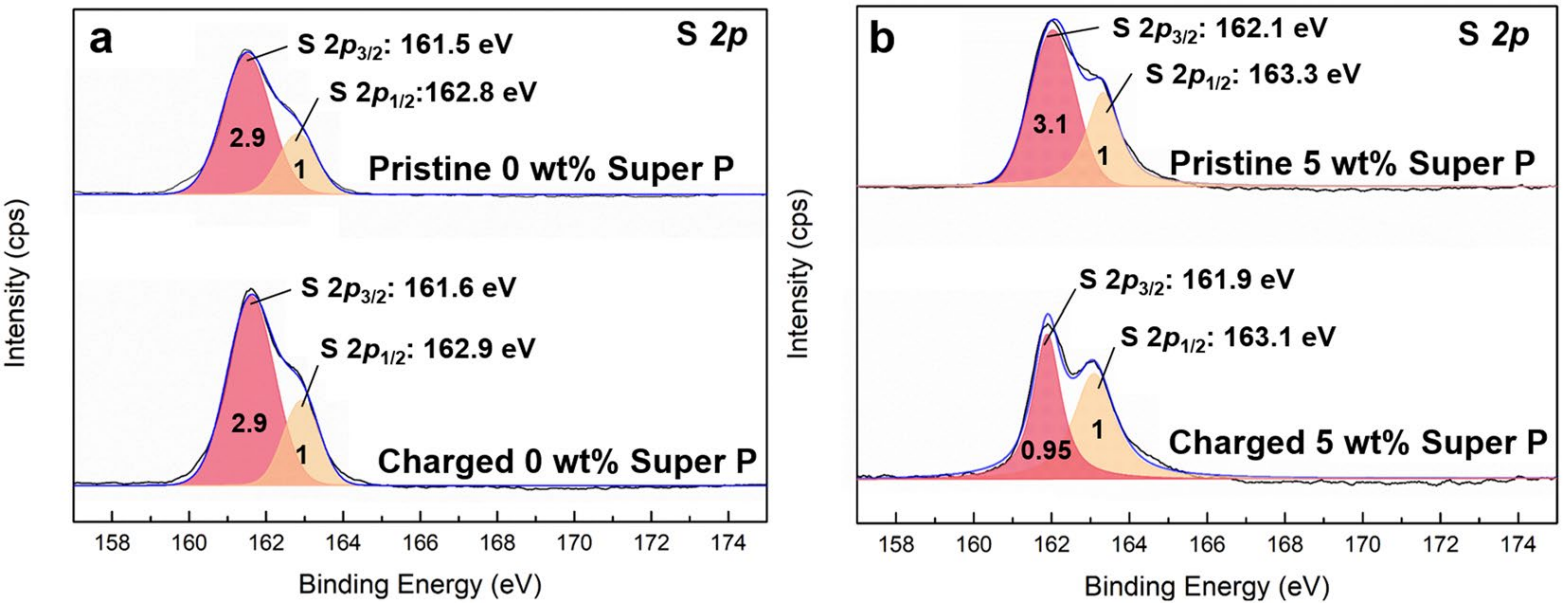
Deconvoluted S *2p* XPS spectra of composite cathodes. (**a**) Top panel: Deconvoluted S *2p* XPS spectra of pristine carbon-free composite cathode. Bottom panel: Deconvoluted S *2p* XPS spectra of first charged carbon-free composite cathode. (**b**) Top panel: Deconvoluted S *2p* XPS spectra of pristine 5 wt% carbon-containing composite cathode. Bottom panel: Deconvoluted S *2p* XPS spectra of first charged 5 wt% carbon-containing composite cathode.

In order to confirm the chemical change of the LGPS triggered by the presence of the carbon in the cathode interface, four samples of composite cathodes were analyzed through surface-sensitive X-ray Photoelectron Spectroscopy (XPS): carbon-free composite cathodes before and after the charge, and 5 wt% Super P-containing composite cathodes before and after the charge, as illustrated in Fig. 4. The fitting of the XPS spectrum of the pristine composite cathode results in roughly two main peaks at ~163 and ~161.5 eV, denoted as a high binding energy (B. E.) peak and a low B.E. peak, respectively. Although the precise designation of each peak is rather challenging due to the complicated chemical environment of S in the sample, the electronegativity difference of cations that are bonded with S could tell the general trend of the binding energy positions (S-S > P-S > Ge-S > Li-S)^31^. The intensity ratio between low B.E. peak and high B.E. peak is 2.9:1 for pristine composite cathode. After the charge process, it is observed that the ratio is maintained along with the same peak positions, indicating that the electrochemical cycling does not notably degrade the LGPS in the carbon-free composite cathode. The pristine 5 wt% carbon-containing composite cathode similarly displays two main peaks at ~163.3 and 162.1 eV with the intensity ratio of 3.1:1 comparable to that of the pristine carbon-free cathode. On the other hand, after the charge process, it notably decreases from 3.1:1 to 0.95:1 as presented in Fig. 4b, revealing that LGPS undergoes a significant change in the structure particularly on the surface, considering the nature of the surface-sensitive XPS. Such a change with suppressed low B.E. peak and the increased high B.E. peak of sulfur suggests that the oxidation of sulfur occurred in the LGPS. Moreover, considering the fact that the low B.E peak can be primarily attributed to Li-S due to the large lithium composition in LGPS (Li_10_GeP_2_S_12_), it proposes that a delithiation process has substantially taken place at the surface of LGPS with charging, which is probably followed by the formation of new S-S bonds (High B.E. peak). This speculation is consistent with the previous observation on the electrochemical reaction of LGPS as an active electrode material where lithium extraction was found to accompany with the formation of S-S bonding upon charging^31^. However, as the reversibility of the LGPS as an electrode was limited to only several cycles, its degradation is expected with the extended cycle numbers^31^. In addition, XPS spectra on C element for pristine 5 wt% Super P composite cathode and charged 5 wt% Super P composite cathode were compared. According to Figure S6, additional or disappearance of the carbon peak for pristine 5 wt% Super P composite cathode was not observed after charge, implying that the carbon simply plays a role of an agent to promote the degradation of LGPS, not directly participating the side reactions.

**Figure 5:**
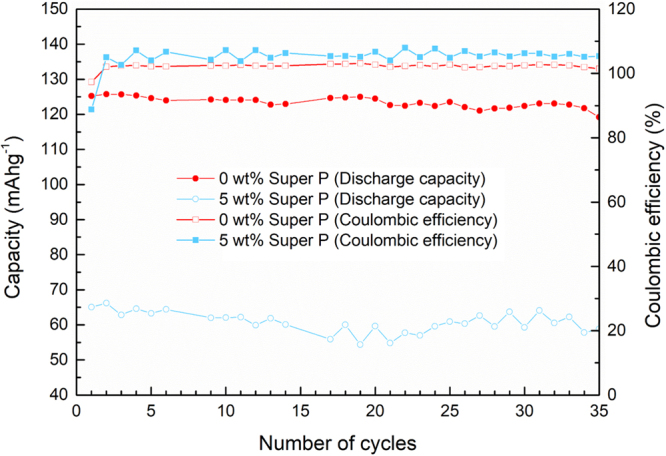
Cycle performance of all-solid-state battery with/without carbon additives in composite cathode. The ASSB with carbon additive (Super P) shows not only decreased initial discharge capacity but also unstable cycle life of ASSB compared to carbon-free ASSB. While capacity of carbon-free ASSB is steadily maintained about 125 mAh g^−1^, capacity of 5 wt% Super P-containing ASSB is unstable throughout 35 cycles.

The participation of LGPS in the electrochemical redox reaction observed in the initial cycle also influences on the cycle life of carbon-containing composite cathodes. Figure 5 shows the capacity retentions of composite cathodes with 0 wt% and 5 wt% Super P. The carbon-free cathode maintains 85% of the theoretical capacity of LiCoO_2_ after 35 cycles under the current rate of 0.075 C. On the other hand, the carbon-containing cathode exhibits substantially low capacity along with the coulombic efficiency that far deviates from 100%, delivering 43% of theoretical capacity of LiCoO_2_ throughout 35 cycles. The slight fluctuation of the discharge capacity throughout 35 cycles is attributed to the decomposition products of LGPS that destabilizes the transport of ions at the interface. The formation and accumulation of side products from LGPS at the interface are expected to hinder Li^+^ transfer, which would increase the impedance of the cell. Furthermore, compared with the pristine LGPS, side products from the decomposition may have different mechanical properties such as ductility^13^. The transformed physical property may not be ductile enough to withstand the stress applied to the cell. While the overall discharge capacity is substantially low, the relative cycle degradation is not as severe as expected. It is speculated that the degradation of LGPS mainly takes place at the initial cycle near the carbon hampering the ionic transport, however, it does not propagate to further influence on the transport, and the degradation of LGPS that is originally far from the carbon remains limited due to the lack of electrical contact. The disappearance of the additional slope after the second cycle also supports that the major decomposition of LGPS occurs at the first charging step (Figure S4).

## Discussion

Introduction of carbon additives in composite cathode induces a large polarization during charging and discharging, followed by the decreased initial capacity along with an unusual voltage slope at around 2.6 V *vs*. Li/Li^+^. The degradation of the electrochemical performance of the carbon-containing cathode composite is found to be correlated with the severe impedance increase at the cathode interface during cycles. The surface analysis of the composite cathode with carbon added revealed that the oxidation of sulfur occurs in LGPS during charging, involving the loss of the substantial lithium and the formation of sulfur-sulfur bonding, which is indicative of the degradation of LGPS. It is believed that the deterioration of the LGPS with carbon that triggers the electrochemical reaction of LGPS increases the interfacial impedance in the composite cathode by interfering the Li^+^ pathway. The large impedance that has been developed at the interface between the carbon and the solid electrolytes inhibits the lithium ion transport, thus the capacity decreases with higher Super P content, which is accompanied by the larger polarization. This work shows that carbon additives which are crucial for constructing high power density ASSBs, can undermine the electrochemical performance of ASSB employing sulfide electrolytes. As we face a dilemma in constructing ASSB concerning the conductive agent, which provides the electronic pathway but simultaneously promotes the degradation of the electrolyte, optimally balancing them or finding a suitable conductive agent with a minimal influence on the electrolyte is essential in designing high power density ASSBs employing sulfide electrolytes.

## Methods

Li_10_GeP_2_S_12_ was prepared as the previous reports described (Figure S1). ^8^Li_2_S, P_2_S_5_, and GeS_2_ were used as starting materials. These precursors were weighed in the molar ratio of Li_2_S/P_2_S_5_/GeS_2_ = 5/1/1 in an argon filled glove box, sealed in a container filled with argon gas and mixed for 30 minutes using planetary ball miller (Wellcos Corporation, NBK-1, Korea). The mixed powder was pressed into pellets with 26 MPa pressure and synthesized at 550 °C for 8 hours in argon atmosphere. The sample was naturally cooled down to the ambient temperature. The composite cathode was prepared by mixing LiNbO_3_-coated LiCoO_2_, LGPS and conductive agents such as Super P carbon black (Hanwha Chemical, Korea), MWCNT (Hanwha Chemical, Korea), and Denka black (Denka Company, Japan) in various weight percentage (0 wt%, 3 wt% and 5 wt%). The weight ratio of LNB-LCO and LGPS was fixed to 70:30. The three components of composite cathode were hand ground by mortar and pestle for 15 minutes in order to obtain homogeneous mixture. ASSBs were assembled in a home designed cell setting (Figure S2). First, 70 mg of LGPS powder was filled into PEEK cell body with inner diameter of 10 mm and cold pressed with 26 MPa for 30 seconds. Then, 20 mg of prepared composite cathode was filled on one side of LGPS pellet in PEEK cell body and cold pressed with 26 MPa for 30 seconds. Thin indium foil with diameter of 9.5 mm (Alfa Aesar, 99.99%, 0.25 mm in thickness) was attached to the other side of LGPS pellet. And, lithium foil with diameter of 4 mm was placed on top of the indium foil and pressed with 4 MPa for 30 seconds. Lastly, cell casing was applied to ASSB cell in order to apply constant pressure (~15 Nm torque).

Galvanostatic cycling of the cells was carried out in voltage range of 1.9–3.6 V *vs*. In/LiIn, which corresponds to 2.5–4.2 V *vs*. Li^+^/Li. The C-rate was calculated based on the practical capacity of LiCoO_2_, 140 mAh g^−1^, considering Li_0.5_CoO_2_ as a fully charged state. All galvanostatic tests were carried out at current rate of 0.075 C and conducted using a battery test system (WBCS 3000, WonA Tech, Korea). Electrochemical Impedance Spectroscopy (EIS) was carried out after 2 cycles of ASSBs to 3.6 V *vs*. In/LiIn at 0.075 C current rate. EIS was conducted from 3 MHz to 0.05 Hz (Model VSP-300, Bio-Logic Science Instruments, France). In the X-ray Photoelectron Spectroscopy (XPS) measurement, the pass energy was set to 23.5 eV. In order to correct the charging effect, C 1 s signal was set to 284.8 eV for ASSBs^28^. All samples were transferred under argon atmosphere with an air-tight transfer vessel. XPSPeak41 software package was used for the data analysis.

## Electronic supplementary material

supplementary information
