# Author Correction: Investigation on the interface between Li_10_GeP_2_S_12_ electrolyte and carbon conductive agents in all-solid-state lithium battery

**DOI:** 10.1038/s41598-018-28601-9

**Published:** 2018-07-04

**Authors:** Kyungho Yoon, Jung-Joon Kim, Won Mo Seong, Myeong Hwan Lee, Kisuk Kang

**Affiliations:** 1 Department of Materials Science and Engineering, Seoul National University, 1 Gwanak-ro, Gwanak-gu, Seoul, 151-742 Republic of Korea; 2 Samsung SDI, Samsung-ro 130, Yeongton-gu, Suwon-si, Gyeonggi-do, 16678 Republic of Korea; 3 Center for Nanoparticle Research at Institute for Basic Science (IBS), Seoul National University, 1 Gwanak-ro, Gwanak-gu, Seoul, 08826 Korea

Correction to: *Scientific Reports* 10.1038/s41598-018-26101-4, published online 23 May 2018

The authors did not cite a related paper and apologise for this oversight.

In the Introduction,

“Furthermore, it suggests that the nature of LGPS in conventional carbon-containing composite cathodes would vary with electrochemical cycling, which has not been considered in the evaluation of the cathode performance. In this respect, here we carefully study the effect of carbon additives in ASSBs based on LGPS electrolyte. It is found that the inclusion of carbon conductive agents regardless of their physical differences such as carbon nanoparticles and carbon nanotubes in composite cathode results in the inferior performance of the cathode in comparison to that without the carbon additives.”

should read:

“Furthermore, it suggests that the nature of LGPS in conventional carbon-containing composite cathodes would vary with electrochemical cycling, which has not been considered in the evaluation of the cathode performance. Very recently, such problem on the cathode interface in practical ASSB cell configuration has been reported by Zhang *et al*., who showed the adverse effect of carbon inclusion in ASSBs^1^. In this respect, here we carefully study the effect of carbon additives in ASSBs based on LGPS electrolyte. It is found that the inclusion of carbon conductive agents regardless of their physical differences such as carbon nanoparticles and carbon nanotubes in composite cathode results in the inferior performance of the cathode in comparison to that without the carbon additives.”
