# Supplementary material for: Investigation on the interface between Li10GeP2S12 electrolyte and carbon conductive agents in all-solid-state lithium battery
Source: Sci Rep. 2018 May 23;8:8066. doi: 10.1038/s41598-018-26101-4 (PMC5966405; doi:10.1038/s41598-018-26101-4)
Supplement: Supplementary file 1 — supplementary information [file 41598_2018_26101_MOESM1_ESM.pdf]

Investigation on the interface between $Li_{10}GeP_2S_{12}$ electrolyte and carbon conductive agents in all-solid-state lithium battery

Kyungho Yoon[1], Jung-Joon Kim[2], Won Mo Seong[1], Myeong Hwan Lee[1], and Kisuk Kang[1, 3, *]

[1]Department of Materials Science and Engineering, Seoul National University, 1 Gwanak-ro, Gwanak-gu, Seoul 151-742, Republic of Korea

[2]Samsung SDI, Samsung-ro 130, Yeongton-gu, Suwon-si, Gyeonggi-do, 16678, Republic of Korea

[3]Center for Nanoparticle Research at Institute for Basic Science (IBS), Seoul National University, 1 Gwanak-ro, Gwanak-gu, Seoul 08826, Korea

* Corresponding author: matlgen1@snu.ac.kr

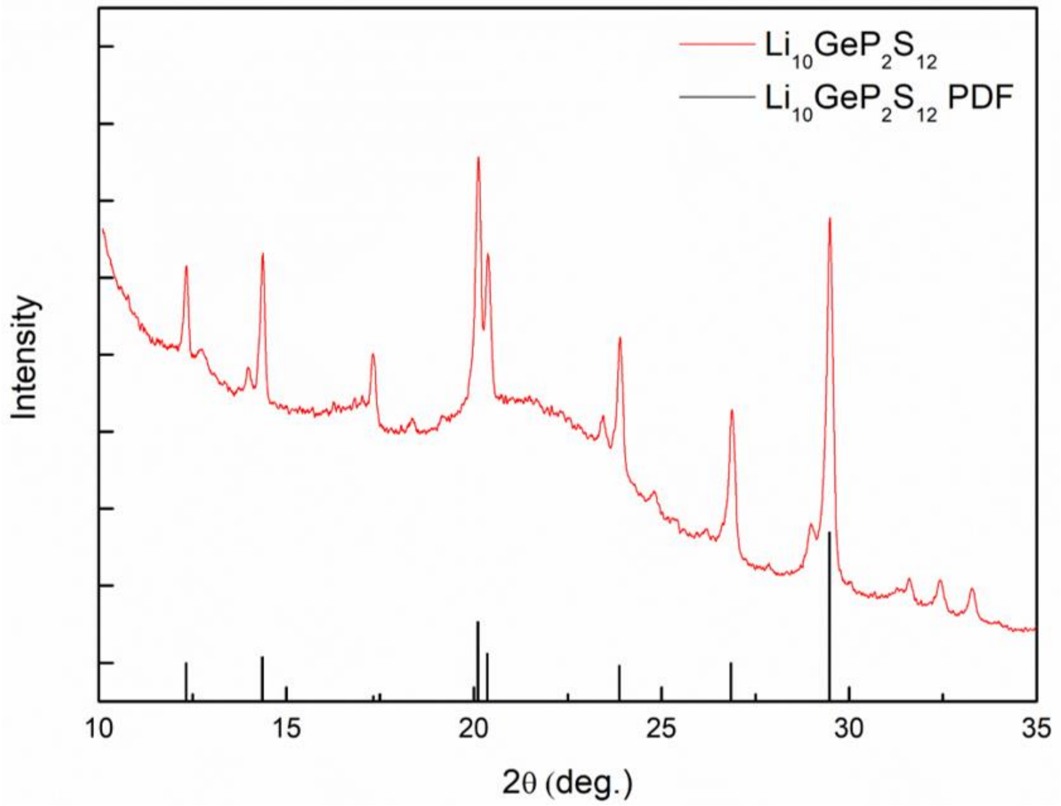

Figure S1. X-Ray Diffraction (XRD) pattern of LGPS solid electrolyte. XRD peaks of LGPS indicates that LGPS is successfully synthesized without side products.

$Li_{10}GeP_2S_{12}$	
Temp (°C)	Conductivity (mS cm^{-1})
0	1.90
25	6.99
80	16.72

Table S1. Calculated ionic conductivity of LGPS. Table S1 shows that the ionic conductivity of LGPS in room temperature is close to the reported value in previous research.[8,28]

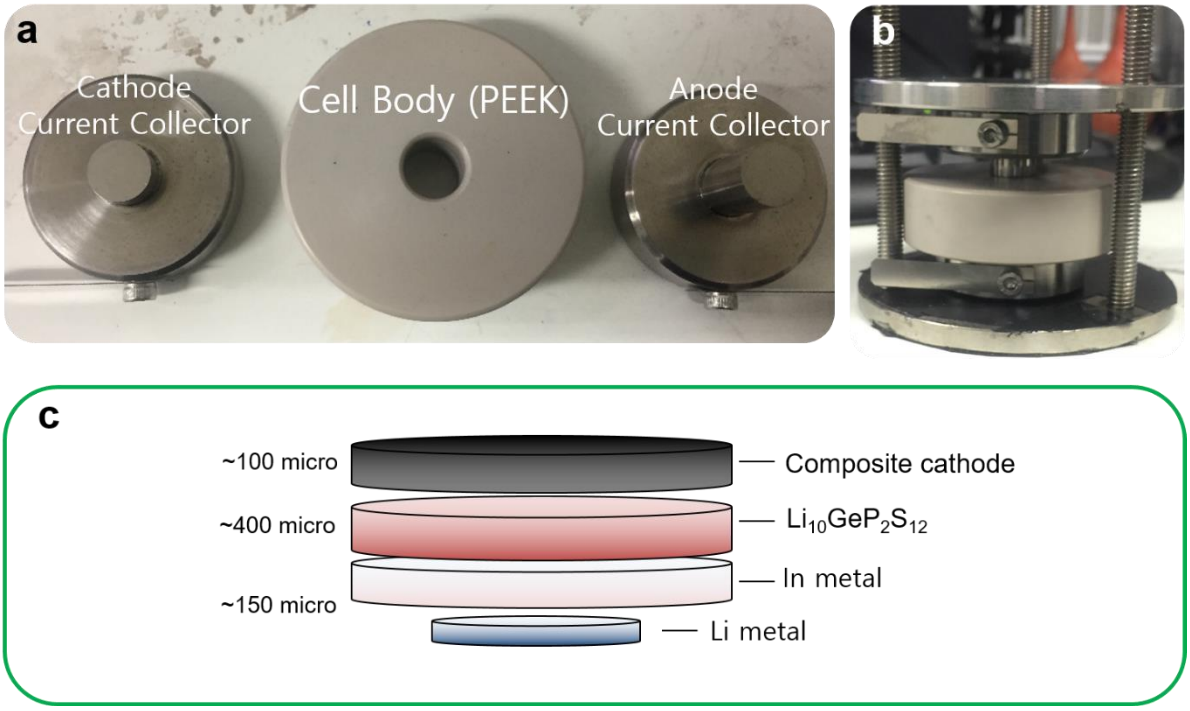

Figure S2. Images and schematic of ASSB employed in this work. a, Image of disassembled ASSB cell. **b,** Image of assembled ASSB cell with cell casing. **c,** Schematic of ASSB pellet inside the cell body (PEEK).

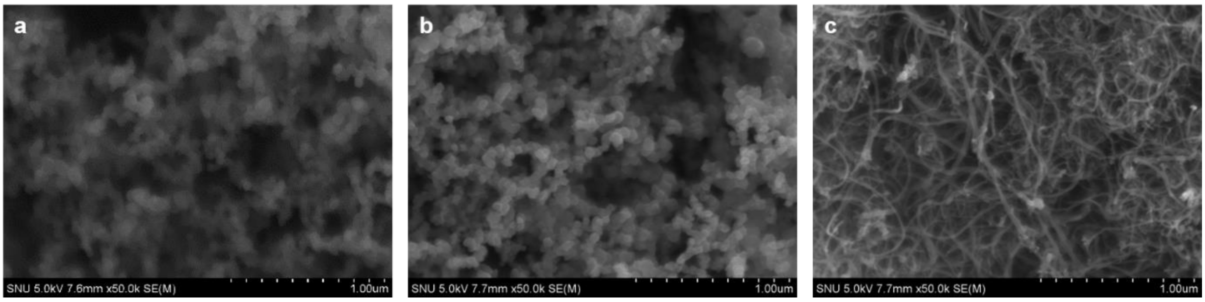

Figure S3. Scanning Electron Microscope (SEM) images for different types of carbon additives. a, SEM image of Super P. b, SEM image of Denka Black. c, SEM image of Multi-Walled Carbon Nanotubes (MWCNT).

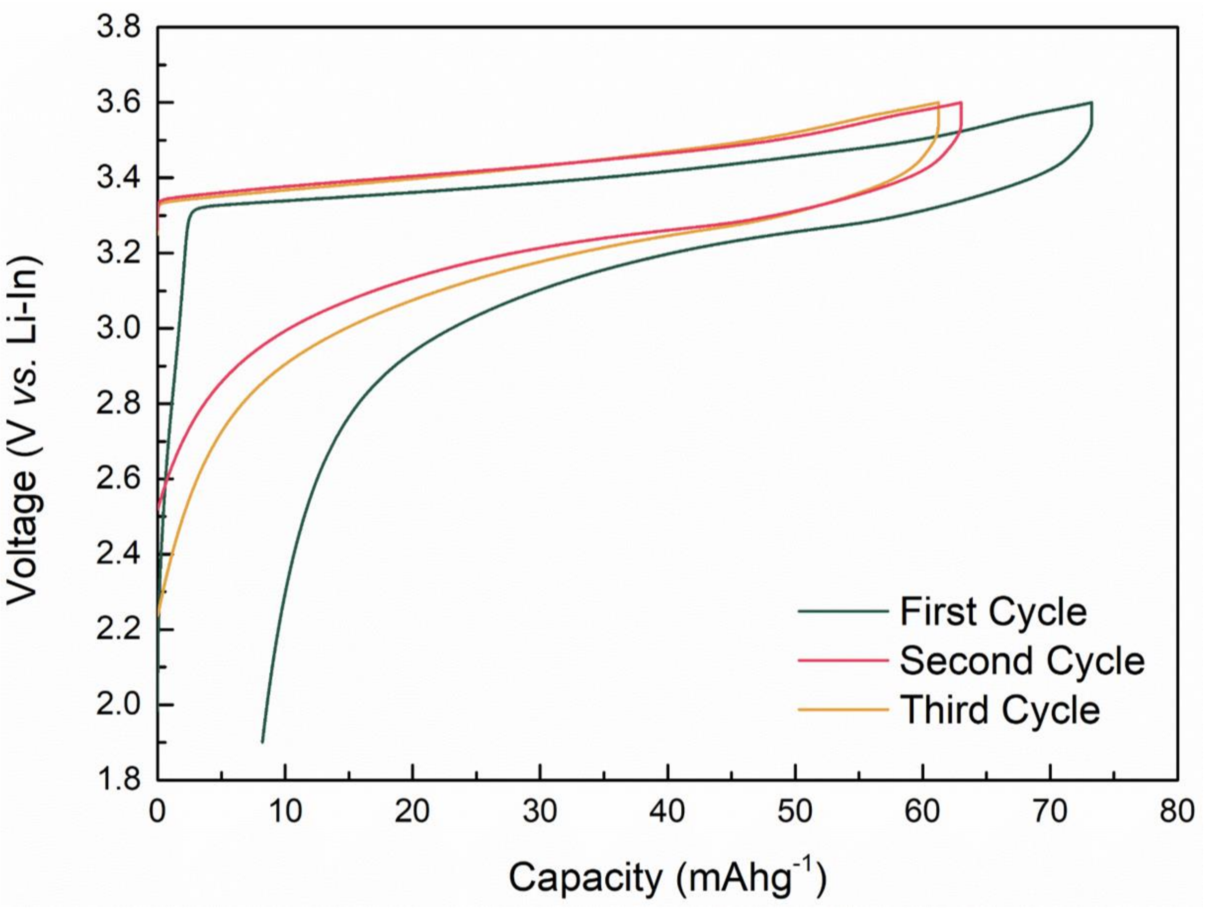

Figure S4. Electrochemical profile of 5 wt% Super P-containing ASSB. Electrochemical profile of 5 wt% Super P-containing ASSB for initial three cycles is shown. The additional slope in charging step is only observed in first charging process.

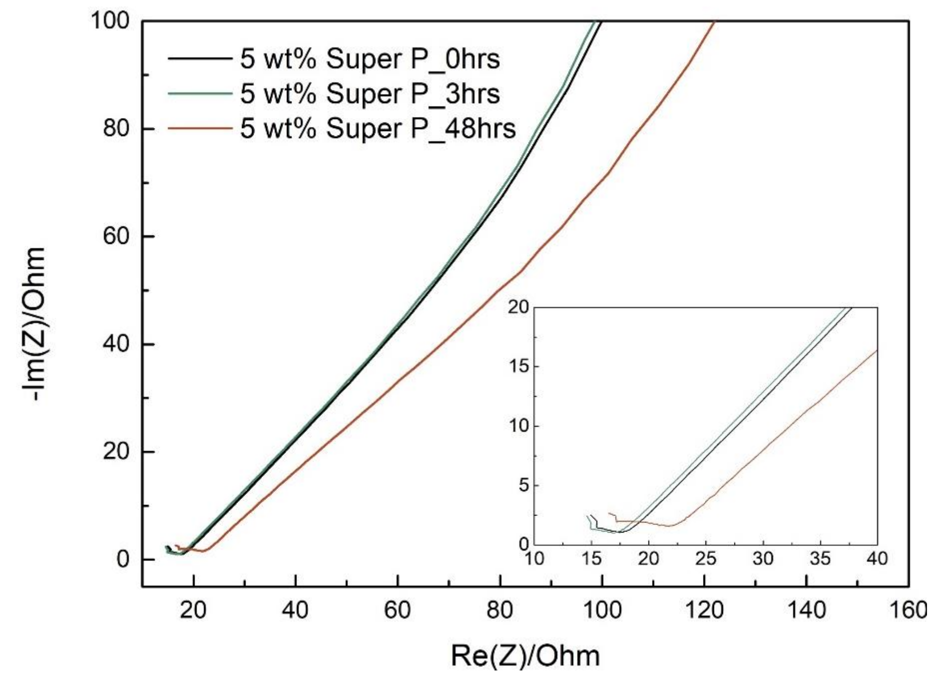

Figure S5. Electrochemical impedance spectra of 5 wt% Super P containing ASSB after various rest time. The inset graph is the magnification of initial electrochemical impedance spectra of ASSB with 5 wt% Super P.

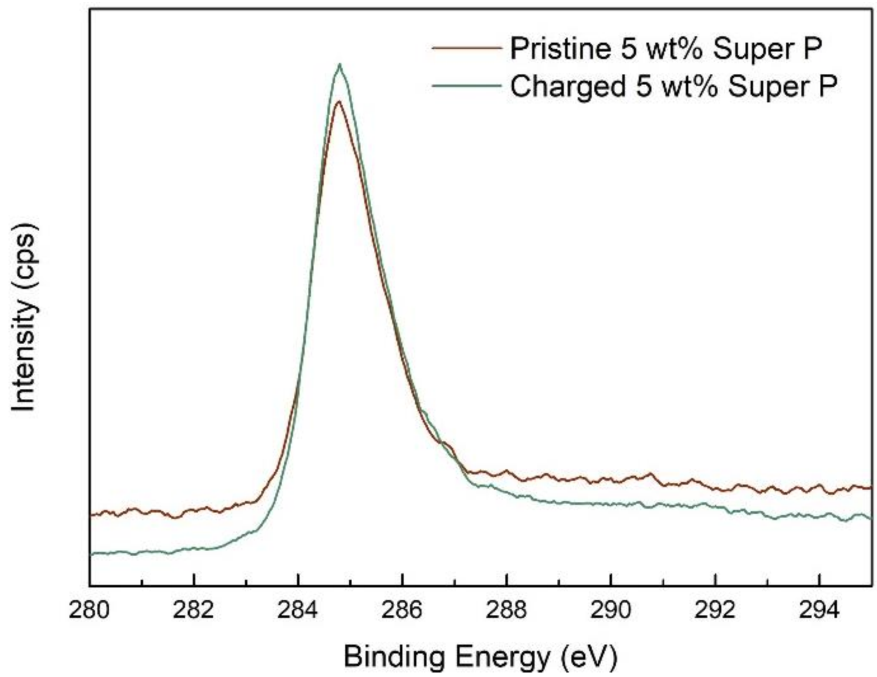

Figure S6. Carbon XPS spectra of composite cathode with 5 wt% Super P before and after charging process.
